# Antioxidative Effects of Carrot-Derived Nanovesicles in Cardiomyoblast and Neuroblastoma Cells

**DOI:** 10.3390/pharmaceutics13081203

**Published:** 2021-08-05

**Authors:** Do Kyung Kim, Won Jong Rhee

**Affiliations:** 1 Department of Bioengineering and Nano-Bioengineering, Incheon National University, Incheon 22012, Korea; ehrud2114@gmail.com; 2 Division of Bioengineering, Incheon National University, Incheon 22012, Korea

**Keywords:** oxidative stress, plant-derived nanovesicle, apoptosis, extracellular vesicle, myocardial infarction, Parkinson’s disease

## Abstract

Oxidative stress is implicated in many diseases, including cardiovascular and neurodegenerative diseases. Because an increased level of oxidative stress causes apoptosis, it is necessary to inhibit cellular responses to oxidative stress. In this study, Carex, a nanovesicle from carrot, was isolated and investigated as a novel biomaterial with antioxidative function in cardiomyoblasts and neuroblastoma cells. A high concentration of nanovesicles was purified from carrots, using size-exclusion chromatography in combination with ultrafiltration. The characterization of Carex demonstrated that it had properties similar to those of extracellular vesicles. Carex showed low cytotoxicity in both H9C2 cardiomyoblasts and SH-SY5Y neuroblastoma cells, when a high level of Carex was delivered to the cells. Carex was further investigated for its antioxidative and apoptotic effects, and it significantly inhibited ROS generation and apoptosis in vitro in myocardial infarction and Parkinson’s disease models. Carex inhibited the reduction of antioxidative molecule expression, including Nrf-2, HO-1, and NQO-1, in both models. Considering its antioxidative function and high production yield, Carex is a potential drug candidate for the treatment of myocardial infarction as well as Parkinson’s disease. Thus, the results demonstrated in this study will contribute to an exploration of a novel drug, using nanovesicles from plants, including carrots.

## 1. Introduction

Reactive oxygen species (ROS) are produced by extrinsic and intrinsic factors and participate in cellular signaling pathways [1]. Oxidative stress occurs due to an imbalance between ROS generation and the cellular antioxidant response. Excessive ROS levels result in the mitochondria membrane potential loss and damage to the cell membrane, DNA, proteins, and lipids [2,3,4]. Previously, it was reported that oxidative stress plays an important role in the initiation and progression of many diseases, including cardiovascular disease, neurodegeneration, and diabetes [5,6,7]. For instance, myocardial hypoxia/reoxygenation causes increased oxidative stress in myocardial tissues, which causes cardiovascular diseases, such as cardiac hypertrophy, cardiomyocyte apoptosis, and heart failure [8]. Oxidative stress in the degeneration of dopaminergic neurons also leads to Parkinson’s disease [9]. Therefore, the development of biomaterials that can inhibit oxidative stress is important for developing efficient drugs that can prevent cardiovascular and Parkinson’s disease.

Extracellular vesicles (EVs), which are 50–1000 nm in size, are produced by cells, including animals, humans, microbes, and plants [10,11,12,13]. In humans, EVs participate in intercellular communication by transferring biologically active cargoes, including proteins, RNA, DNA, and lipids [14,15,16,17,18]. In this context, EVs produced from mammalian cell cultures have been extensively studied as potential therapeutics or drug delivery vehicles for disease treatment [19,20,21,22,23,24,25]. However, there are crucial limitations to using cell-culture-derived EVs. Because a large number of cell-culture-derived EVs are necessary for clinical application, they require high production costs and time to produce a large amount of EVs. Although some cell-culture-derived EVs have shown certain therapeutic effects, including tissue repair [19,20,26], they have limited biological activities in most cases. Thus, the engineering of EVs by encapsulating pharmaceutical drugs, such as miRNA, chemicals, peptides, and proteins into EVs, is required to endow their therapeutic activities [27,28,29,30]. Furthermore, mammalian cell cultures accompany the use of animal-derived materials, including fetal bovine serum, which is commonly prohibited in drug approval, due to safety issues. Thus, it is necessary to develop alternative sources of EV production to overcome these issues.

Recently, EVs from plants have gained considerable attention as potential therapeutic agents, owing to their anti-cancer [31,32,33], antioxidative [34,35], and anti-inflammatory potential [14,36,37,38,39]. Plant-derived nanovesicles showed high biocompatibility and low toxicity [14,40]. Previous evidence suggests that plant-derived nanovesicles can enter mammalian cells and serve as cross-species messengers [41,42]. To date, however, there are a limited number of studies exploring the biological activities of plant-derived nanovesicles in various human disease models.

**Figure 1:**
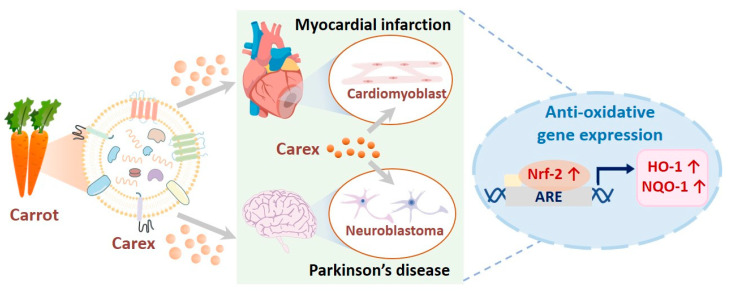
Illustration of carrot-derived nanovesicle (Carex) isolation from carrots and the investigation of the antioxidative effects and molecular regulations in cardiomyoblast and neuroblastoma cells as in vitro model systems for myocardial infarction and Parkinson’s disease, respectively.

Carrots were introduced as novel sources of plant-derived nanovesicles because they are widely and easily cultivated, and thus, carrot-derived EVs (Carex) were investigated for their biological activities. To achieve this, Carex was isolated from carrots, using size-exclusion chromatography in combination with ultrafiltration. In addition, their antioxidative properties and regulation of antioxidative molecules in heart-derived cardiomyoblasts and neuroblastoma cells as in vitro disease models were explored (Figure 1). Considering its strong antioxidative activity and high productivity, Carex can be suggested as a novel biomaterial that can be widely applied as a therapeutic drug for diseases, including myocardial infarction and Parkinson’s disease.

## 2. Materials and Methods

### 2.1. Cell Culture and Treatment

H9C2 embryonic rat heart-derived cardiomyoblasts and human neuroblastoma SH-SY5Y cells were obtained from the Korean Cell Line Bank (Seoul, Korea). H9C2 and SH-SY5Y cells were cultured in Dulbecco’s modified Eagle’s medium (DMEM; Corning Inc., NY, USA), DMEM-F12 medium supplemented with 10% FBS (Gibco, Waltham, MA, USA), and penicillin and streptomycin (Gibco, NY, USA). All the cells were cultured in 5% CO_2_ at 37 °C. Hydrogen peroxide (H_2_O_2_; Sigma-Aldrich, St. Louis, MO, USA) was used to induce oxidative stress in H9C2 cells. 6-Hydroxydopamine hydrochloride (6-OHDA; Sigma-Aldrich, MO, USA) was used as an oxidative stress inducer in SH-SY5Y cells.

### 2.2. Isolation of Carex from Carrot

For Carex isolation, carrots (*Daucus carota* subsp. *Sativus*) were purchased from local farms in the Republic of Korea. The whole carrot was washed with distilled water for the removal of soil, dust, and pesticides. The carrot juice was produced by extraction using a blender, followed by serial centrifugation at 8000 and 20,000× *g* for 1 h each for large debris removal. The juice was stored at −80 °C before isolation. An Amicon Ultra-15 filter unit (Millipore, Burlington, MA, USA) was used for the juice concentration, followed by size-exclusion chromatography for Carex isolation (Izon Science, Christchurch, New Zealand). Because PBS preserves EVs’ functionality and integrity, PBS was used for size-exclusion chromatography. Each fraction was eluted, and the nanovesicles and protein concentrations of each fraction were assessed.

### 2.3. Characterization of EVs

The Carex concentration and size distribution were measured, using nanoparticle tracking analysis (NS300, Malvern Panalytical, Malvern, UK). The same camera level and threshold were used for all experiments. For transmission electron microscopy (TEM) imaging, the sample was applied to copper grids coated with a thin carbon foil (Ted Pella, Inc., Redding, CA, USA). After allowing the sample to absorb and blotting off the buffer solution onto Whatman paper, the sample on the grids was stained with 2% (*w*/*v*) uranyl acetate for 1 min. Then, distilled water was added for 1 min to remove the uranyl acetate, followed by drying for 15 min. The images were recorded using a Bio-High voltage EM system (JEOL Ltd., Tokyo, Japan). The polydispersity index (PDI) and zeta potential were measured, using DLS (Zetasizer NS, Malvern Panalytical, Malvern, UK) at 25 °C.

### 2.4. Intracellular Uptake of Carex

To find that Carex can be taken up by mammalian cells, Carex was stained with PKH67 green dye (Sigma-Aldrich, MO, USA) for 15 min at 25 °C or DiI dye (Thermo Scientific, Waltham, MA, USA) for 15 min at 37 °C. The labeled Carex was filtered by ultrafiltration (100-kDa) to remove the free dye. H9C2 cells were cultured in a medium with PKH67-labeled Carex at a concentration of 1.0 × 10^11^ particles/mL. Hoechst 33342 fluorescent dye (Cell Signaling Technology, Danvers, MA, USA) was added to the medium for nuclei staining. Cells were washed several times and observed, using a fluorescence microscope (Nikon Corp., Minato, Japan). The DiI dye-labeled Carex uptake was analyzed using a flow cytometer (Beckman Coulter, Inc., Brea, CA, USA).

### 2.5. Intracellular ROS Generation Measurement

H9C2 cells were pre-treated with Carex (1.0 × 10^11^ particles/mL) for 24 h followed by 500 μM H_2_O_2_ treatment for 3 h. Intercellular ROS levels were detected using 2′,7′-dichlorodihydrofluorescein diacetate (H2DCFDA; Thermo Scientific, MA, USA) staining for 1 h at 37 °C. After incubation, the cells were stained with Hoechst 33342 for nuclear staining. The cells were washed with PBS and observed using fluorescence.

### 2.6. Cytotoxicity and Cell Proliferation Assessment

To evaluate the cytotoxicity of Carex, H9C2 and SH-SY5Y cells were treated with Carex at different concentrations ranging from 1.0 × 10^9^ to 1.0 × 10^12^ particles/mL from 48 to 96 h. Cell viability was measured by trypan blue dye staining, and cell proliferation was assessed by a WST-1 assay.

### 2.7. Caspase-3 Activity Measurement

Caspase-3 activity was assessed in H_2_O_2_ or 6-OHDA-treated H9C2 and SH-SY5Y cells, respectively. The cells were washed and lysed with RIPA buffer (ELPIS-Biotech, Korea). The protein concentration was quantified, using a bicinchoninic acid (BCA) assay (Thermo Scientific, MA, USA). The cell lysates were diluted and transferred to black 384-well plates, followed by the addition of an equal volume of Ac-DEVD-AFC (Enzo, Farmingdale, NY, USA)-containing reaction buffer. After incubation, fluorescence intensity was measured at excitation and emission wavelengths of 400 and 505 nm, respectively, using a VarioskanTM Flash Multimode Reader (Thermo Scientific, MA, USA).

### 2.8. Real-Time Quantitative PCR

To assess the effect of Carex on nuclear factor erythroid-2-related factor 2 (Nrf-2) and its target genes, including heme oxygenase 1 (HO-1) and NAD(P)H quinone oxidoreductase 1 (NQO-1), a real-time polymerase chain reaction (RT-PCR) was performed by StepOnePlus Real-Time PCR System (Applied Biosystems, Waltham, MA, USA). H9C2 and SH-SY5Y cells were cultured in the presence or absence of Carex, followed by the induction of oxidative stress by H_2_O_2_ or 6-OHDA. RNA was isolated using the FavorPrepTM Tri-RNA reagent (FAVORGEN Biotech Corp.,Changzhi, Taiwan), and RNA concentrations were assessed using a plate reader (BioTek Instruments, Winooski, VT, USA). cDNA synthesis was performed using ReverTra Ace qPCR RT Master Mix (Toyobo, Osaka, Japan), and reverse transcription-PCR was performed using THUNDERBIRD SYBR qPCR mix (Toyobo, Japan).

### 2.9. Western Blot Analysis

H9C2 cells were supplemented with Carex and incubated with H_2_O_2_ to induce oxidative stress. Cells were lysed in RIPA buffer, and the protein concentration was measured, using the BCA assay. Forty micrograms of protein were separated by SDS-PAGE. The proteins were then transferred to a PVDF membrane at 70 V for 2 h. After blocking with 5% skim milk, the membranes were then incubated with Nrf-2 (Cell Signaling Technology, MA, USA), HO-1 (Cell Signaling Technology, MA, USA), and GAPDH (Cell Signaling Technology, MA, USA) antibodies overnight at 4 °C. After washing, an anti-rabbit IgG with horseradish peroxidase (Cell Signaling Technology, USA) was incubated. ECL Blotting Reagent (Cytiva, Marlborough, MA, USA) was used for the chemiluminescence reaction, followed by analysis using the ChemiDoc™ XRS System (Bio-Rad, Hercules, CA, USA).

## 3. Results

### 3.1. Nanovesicle Isolation from Carrot Using Ultrafiltration and Size-Exclusion Chromatography

To investigate the biomedical applications of Carex, it is essential to produce nanovesicles from carrots with high yield and purity. Although polyethylene glycol-based precipitation methods and ultracentrifugation are widely used for nanovesicle isolation, both methods have critical defects. First, precipitation methods are criticized because they also co-precipitate high amount of protein impurities [14,43]. Ultracentrifugation provides low yields, as a large number of EVs are lost during centrifugation. In addition, nanovesicles can be disrupted during isolation due to high centrifugal forces, which may complicate the use of isolated nanovesicles as therapeutic drugs [23,44,45]. In our previous study, nanovesicles from cabbages were successfully isolated using size-exclusion chromatography combined with ultrafiltration [14]. Thus, the same isolation strategy was adopted for nanovesicle isolation from carrots.

**Figure 2:**
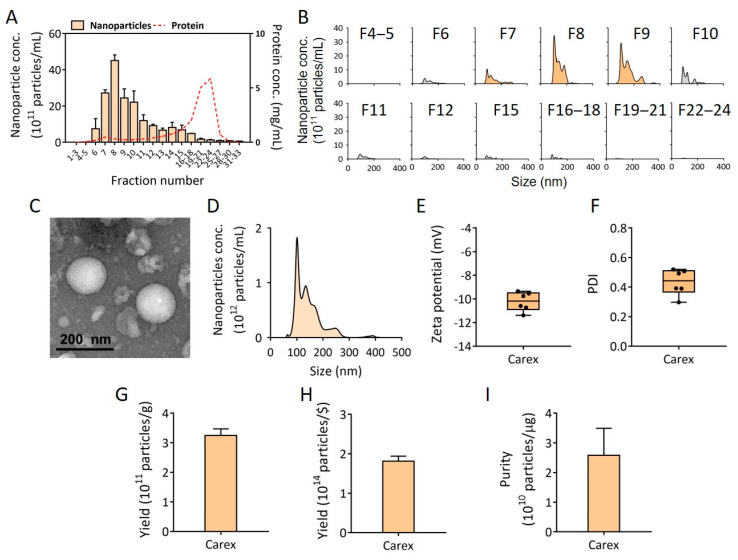
Isolation of nanovesicles from carrots and its characterization. (**A**) Carex was isolated and purified from carrots, using ultrafiltration followed by size-exclusion chromatography. The concentrations of nanovesicles and proteins in all size-exclusion chromatography fractions were assessed. (**B**) The size distribution of each fraction was shown. The nanovesicle-containing fractions (fraction 7–9) were used for further characterization. (**C**) The morphology of Carex was analyzed using TEM. (**D**) The representative size distribution of Carex was analyzed using NTA. (**E**,**F**) The zeta potential and PDI of Carex were analyzed using DLS. (**G**–**I**) Carex yield per g of carrot (**G**), yield per carrot price, and purity per μg of protein impurities are shown.

Due to the limited injection volume for size-exclusion chromatography, carrot juice with nanovesicles was concentrated, using ultrafiltration. Then, the concentrated juice was injected into a size-exclusion chromatography column for nanovesicle isolation. The concentrations and size distributions of nanovesicles in each fraction (33 fractions total) were measured using NTA, and the impurity (protein) concentrations in each fraction were also assessed. As shown in Figure 2A,B, a high concentration of nanovesicles was collected in fractions from 7 to 9, while most protein impurities were separated in fractions from 13 to 30, indicating that nanovesicles were successfully isolated and separated from the protein contaminants from carrots. The fractions from 7 to 9 were collected as Carex and further characterized for the experiments.

### 3.2. Characterization of Isolated Carex from Carrots

Carex from carrots was characterized for its biophysical properties after isolation using size-exclusion chromatography. First, the morphology of Carex was observed, using TEM. Carex showed a spherical shape with an average size of approximately 150 nm (Figure 2C). The size distribution of Carex was analyzed using the NTA (Figure 2D). Nanovesicles showed several peaks, meaning that there were heterogeneous nanoparticles, and the average size of Carex was 143.9 nm, which is within the range of the known sizes of EVs (Figure 2D). The zeta potentials and PDI of Carex were further characterized (Figure 2E,F). The average zeta potential of Carex was −10.2 mV, indicating that it was negatively charged, and the PDI value was 0.43.

To be widely harnessed as a commercial therapeutic, the high productivity of Carex from carrot is essential. The high yields based on the weight as well as the price of carrots can be hugely beneficial because the production of EVs from cell culture requires scale-up culture, high cost, and long production time. To calculate the final yield of Carex, the fractions from size-exclusion chromatography (fraction 7–9) containing Carex were mixed together (totally 1.5 mL), and the Carex concentration was measured using NTA. Then, the absolute number of Carex was divided by grams of carrots used for carrot juice production to obtain the number of Carex per gram of carrots. It is noteworthy that the number of Carex per gram of carrots was 3.24 × 10^11^ particles/g (Figure 2G), which is equal to 1.81 × 10^14^ particles/$ (USD) (Figure 2H). Considering that the Carex concentration used in the following experiments was 10^11^ particles/mL culture medium, only 0.55% (USD) was required for the preparation of 1 L of culture medium. This is meaningful because the administration of EVs in human clinical trials requires a large amount of EVs. The yield was even higher than those from other plants, including cabbage (1.504 × 10^11^ particles/g) and red cabbages (1.098 × 10^11^ particles/g) [14]. The purity of Carex (particles/μg protein) was also evaluated by dividing the Carex particle concentration by the protein impurity concentrations in Carex fractions (Figure 2I). As a result, the purity of Carex was 2.58 × 10^10^ particles/μg. Based on the previous results using cabbage and red cabbage as nanovesicles sources [14], Carex resulted in higher purity than those of Cabex (1 × 10^10^ particles/μg, cabbage nanovesicles) and Rabex (2 × 10^10^ particles/μg, red cabbage nanovesicles).

### 3.3. Cytotoxicity and Intracellular Delivery of Carex in H9C2 Heart-Derived Cardiomyoblasts

**Figure 3:**
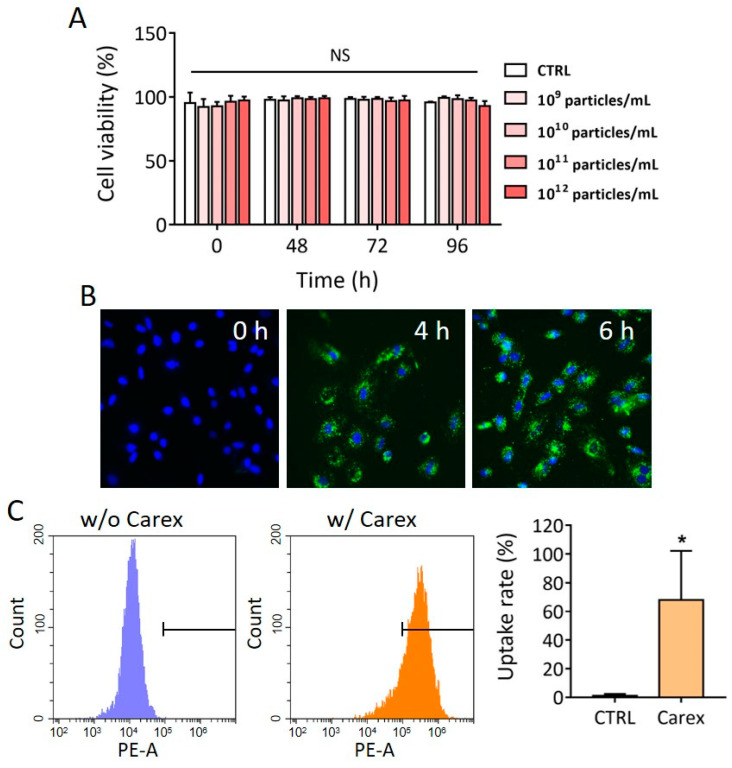
Cytotoxicity and intracellular uptake of Carex in H9C2 cardiomyoblast. (**A**) H9C2 cells were supplemented with Carex, and cell viability was assessed at 0, 48, 72, and 96 h. Note that no cytotoxicity was observed regardless of the Carex dosages. (**B**,**C**) Fluorescence microscopic analysis (**B**) and flow cytometry (**C**) of intracellular delivery of Carex. Carex was stained with PKH67 dye (**B**) or DiI dye (**C**) and supplemented to the culture medium. Cells were further cultured for 4 and 6 h for fluorescence microscopic analysis and 4 h for flow cytometric analysis. The uptake rate (%) was calculated from the flow cytometric analysis results. All values are expressed as mean ± SD (* *p* < 0.05, NS: not significant; *n* = 3).

Before we investigated the antioxidative effect of Carex in mammalian cells, cytotoxicity was observed by supplementing different concentrations of Carex (10^9^–10^12^ particles/mL) into the culture medium. H9C2 heart-derived cells were cultured in the presence of Carex, and cell viability was measured for 96 h using the WST-1 assay. No decrease in cell viability was observed, regardless of Carex concentration (Figure 3A). Although supplementation with the highest dose (10^12^ particles/mL) of Carex showed no difference in cell viability, 10^11^ particles/mL of Carex was chosen as the final treatment dose for the rest of the experiments. It was previously reported that EVs can be uptaken by cells through different mechanisms, including clathrin-mediated endocytosis, macropinocytosis, lipid raft-mediated endocytosis, caveolin-mediated endocytosis, and plasma membrane fusion [46]. The uptake of Carex by mammalian cells was further investigated. This is an important process because Carex should first interact with cells and deliver biomolecules to cells to exert effects. To demonstrate the nanovesicle penetration to cells, isolated EVs were labeled with PKH67 dye or DiI dye, followed by the removal of unstained free dyes. H9C2 heart-derived cells were cultured with a medium supplemented with 1.0 × 10^11^ particles/mL of labeled Carex. High fluorescence was observed by fluorescence microscopy, indicating that PKH67 dye-labeled Carex was taken up by the cells (Figure 3B). The fluorescence intensities gradually increased as the incubation time increased. Quantitative analysis of cellular uptake of Carex was also performed, using a flow cytometer. As shown in Figure 3C, a large shift in the fluorescence signal was observed when the cells were incubated with DiI dye-labeled Carex. A total of 68.0% cells were observed to take up Carex within 4 h. Thus, Carex was capable of transferring its biomolecules to mammalian cells, which subsequently regulated the biological activities of target cells.

### 3.4. Anti-Oxidative Effect of Carex in H9C2 Heart-Derived Cardiomyoblasts

**Figure 4:**
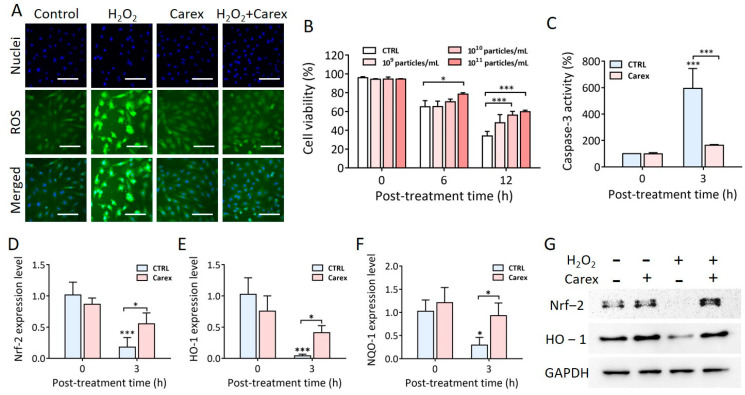
Antioxidative and apoptotic effect of Carex in H9C2 cardiomyoblasts. (**A**) Cells were supplemented with 1 × 10^11^ particles/mL of Carex by oxidative stress induction, using H_2_O_2_. The intracellular ROS levels (green) and nuclei (blue) were stained using H2DCFDA and Hoechst 33342, respectively, followed by fluorescence microscopic observation. The scale bars indicate 100 μm. (**B**) Cells were supplemented with different concentrations of Carex for 1 d followed by H_2_O_2_ treatment. Cell viability was measured by a WST-1 assay. (**C**) Caspase-3 inhibition in H9C2 cells by Carex. (**D**–**F**) RT-PCR analysis of Nrf-2 (**D**), HO-1 (**E**), and NQO-1 (**F**) mRNA expression levels. (**G**) Western blot analysis of Nrf-2 and HO-1 protein expression levels. GAPDH was used as an internal control. All values are expressed as mean ± SD (* *p* < 0.05, *** *p* < 0.001; *n* = 3).

As mentioned earlier, the increased level of oxidative stress due to myocardial hypoxia/reoxygenation caused cardiomyocyte apoptosis and heart failure. Thus, it is necessary to effectively protect cells from oxidative stress and prevent apoptosis without inducing cytotoxicity. In this context, Carex was tested for its antioxidative effect in heart-derived cardiomyoblasts. Oxidative stress was induced by H_2_O_2_ treatment of H9C2 cells for 2 h, followed by H2DCFDA staining to measure intracellular ROS levels (Figure 4A). Lower ROS levels were observed in cells cultured in the absence or presence of 1.0 × 10^11^ particles/mL of Carex when cells were not treated with H_2_O_2_. Intracellular ROS levels drastically increased when cells were treated with 500 μM H_2_O_2_ in the absence of Carex. In contrast, no difference was observed when cells were cultured in the presence of Carex, even after H_2_O_2_ treatment. This indicated that Carex successfully suppressed ROS generation in H9C2 heart-derived cells. Time- and dose-dependent inhibition of apoptosis by Carex after H_2_O_2_ treatment was also demonstrated (Figure 4B). Cells were supplemented with different doses of Carex, ranging from 1.0 × 10^9^ to 1.0 × 10^11^ particles/mL, and incubated for 24 h. Cell viability was assessed at 0, 6, and 12 h post H_2_O_2_ treatment. The cell viability gradually decreased when cells were not supplemented with Carex at 65.0% and 33.9% at 6 and 12 h post-treatment, respectively. However, a significant dose-dependent inhibition of apoptosis was observed when cells were incubated with Carex. For instance, cell viability was 47.9%, 56.0%, and 59.8% when cells were supplemented with 1.0 × 10^9^, 1.0 × 10^10^, and 1.0 × 10^11^ particles/mL of Carex, respectively, at 12 h post H_2_O_2_ treatment. The effect of Carex on caspase-3 activation, which is a key process in the progression of apoptosis, was further analyzed. H9C2 cells were cultured in the absence or presence of Carex, and H_2_O_2_ was treated thereafter. Caspase-3 activity was measured in cell lysates 3 h post-H_2_O_2_ treatment (Figure 4C). Caspase-3 activity drastically increased to 593.4% after H_2_O_2_ treatment for 3 h. In contrast, caspase-3 activation was significantly inhibited by Carex supplementation, which only increased to 163.3% after H_2_O_2_ treatment. Thus, the results indicated that Carex contains components that can suppress ROS generation and inhibit apoptosis caused by oxidative stress. The result supports the potential use of Carex in the treatment of diseases related to aberrant apoptosis and ROS generation, including myocardial infarction.

### 3.5. Anti-Oxidative Mechanism of Carex in H9C2 Heart-Derived Cardiomyoblasts

To explore the antioxidative molecular mechanism of Carex, the expression of antioxidative molecules was measured. As shown in Figure 1, the Nrf-2 signaling pathway is a well-known pathway, and Nrf-2 is a transcription factor that promotes the expression of antioxidative proteins, including HO-1 and NQO-1, thereby regulating cellular responses to oxidative stress. Consequently, Nrf-2 mRNA levels significantly decreased from 102% to 18% 3 h after H_2_O_2_ treatment in the absence of Carex (Figure 4D). The basal Nrf-2 level was similar in healthy H9C2 cells, regardless of Carex supplementation. However, the decrease in Nrf-2 expression was significantly suppressed when cells were supplemented with Carex after H_2_O_2_ treatment. The Nrf-2 expression level decreased from 87% to 56% in Carex-supplemented cells after H_2_O_2_ treatment. Thus, it is clear that Carex inhibited the decrease in Nrf-2 expression in cardiomyoblast cells, thereby protecting cells from oxidative stress. The downstream regulation of HO-1 and NQO-1 by Nrf-2 was analyzed using RT-PCR (Figure 4E,F). First, the basal levels of HO-1 were almost the same in untreated healthy H9C2 cells in the absence and presence of Carex. After H_2_O_2_ treatment for 3 h, however, the HO-1 expression level dropped to approximately 4% as compared to untreated cells in the absence of Carex. This is due to the decrease in Nrf-2 expression levels in cardiomyoblasts caused by H_2_O_2_ treatment. In contrast, the decrease in HO-1 expression was drastically reduced when cells were supplemented with Carex. Similar expression patterns were observed for NQO-1 expression, indicating that Carex effectively inhibited the decrease in this antioxidative protein expression (Figure 4F). The regulation of antioxidative proteins by Carex was further demonstrated by Western blot analysis. As shown in Figure 4G, the Nrf-2 protein level increased after H_2_O_2_ treatment in H9C2 cells supplemented with Carex, but drastically decreased in the absence of Carex. Additionally, no apparent decrease in HO-1 protein was also observed when cells were supplemented with Carex after H_2_O_2_ treatment. Thus, it is clear that Carex is a novel nanovesicle that suppresses apoptosis caused by oxidative stress by efficiently inhibiting the decrease in antioxidative proteins.

### 3.6. Resistance to Oxidative Stress Induced by 6-Hydroxydopamine-Treated Human Neuroblastoma Cells Supplemented with Carex

As described earlier, Parkinson’s disease is a chronic neurological disease; increased oxidative stress in the degeneration of dopaminergic neurons leads to Parkinson’s disease. Thus, increased resistance to oxidative stress and suppression of apoptosis are essential to overcome the progression of Parkinson’s disease. Because Carex showed strong antioxidative and apoptotic effects, Carex was investigated for its antioxidative effect and the regulation of antioxidative molecules in human neuroblastoma SH-SY5Y cells treated with 6-OHDA, which is widely used as an in vitro Parkinson’s disease model [47].

**Figure 5:**
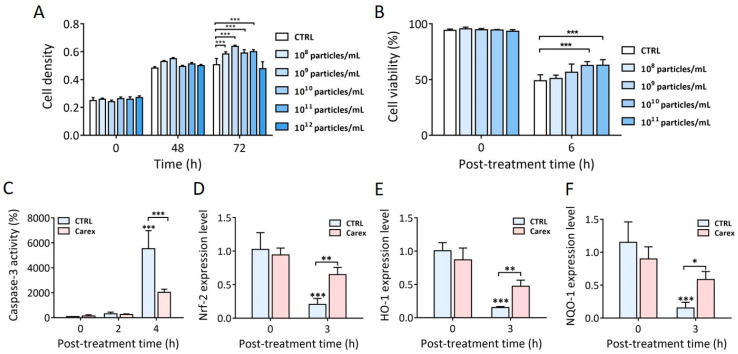
Antioxidative and apoptotic effect of Carex in human neuroblastoma SH-SY5Y cells. (**A**) Carex dose-dependent effect on cell densities and its cytotoxicity was measured. (**B**) Anti-apoptotic effect of Carex in SH-SY5Y cells treated with 6-OHDA using WST-1 assay. (**C**) Inhibition of caspase-3 activity in SH-SY5Y cells supplemented with 1 × 10^11^ particles/mL of Carex. (**D**–**F**) RT-PCR analysis of Nrf-2 (**D**), HO-1 (**E**), and NQO-1 (**F**) mRNA expression levels in SH-SY5Y cells treated with 6-OHDA. All values are expressed as mean ± SD (* *p* < 0.05, ** *p* < 0.01, *** *p* < 0.001; *n* = 3).

First, the dose-dependent cytotoxicity and the effect of Carex on neuroblastoma proliferation were tested (Figure 5A). In general, cell density increased slightly in the presence of Carex. No decrease in cellular density was observed, even for cells supplemented with the highest dose compared to the unsupplemented control. Thus, the results indicated that Carex exhibited a very low level of cytotoxicity. Based on this, further investigation was performed with Carex doses lower than 1 × 10 ^11^ particles/mL.

Then, neuroblastoma SH-SY5Y cells were treated with 200 μM 6-OHDA to induce intracellular oxidative stress, followed by apoptosis (Figure 5B). Cell viability decreased to 48.9% when the cells were treated in the absence of Carex. In contrast, cell viability gradually increased, consistent with the increased Carex doses. As a result, the cell viability was 62.8% when they were supplemented with 1 × 10 ^11^ particles/mL of Carex 6 h after 6-OHDA treatment. The results demonstrated that Carex significantly suppressed neuroblastoma apoptosis caused by increased oxidative stress in cells. To further confirm the effect of Carex, the intracellular levels of caspase-3 activity were assessed in 6-OHDA treated neuroblastoma SH-SY5Y cells. Caspase-3 activity drastically increased 4 h after 6-OHDA treatment in cells without Carex supplementation (Figure 5C). However, supplementation with Carex (1 × 10 ^11^ particles/mL) efficiently inhibited caspase-3 activation. The relative caspase-3 activities were 5527 and 2029% in the absence and presence of Carex, respectively. Thus, it is clear that Carex has an antioxidative effect in neuroblastoma cells in an in vitro Parkinson’s disease model.

The intracellular regulation of antioxidative molecules by Carex was also explored. The Nrf-2 expression level changed from 1.03 to 0.21 (79.6% decrease) in the absence of Carex 3 h after 6-OHDA treatment. However, the Nrf-2 expression level decreased from 0.92 to 0.61 (only 33.7% decrease) in the presence of Carex in the same treatment time (Figure 5D). Changes in the downstream targets of Nrf-2, including HO-1 and NQO-1, were also explored. HO-1 expression level decreased from 1.0 to 0.15 (85% decrease) when cells were treated with 6-OHDA without Carex supplementation. However, the level decreased from 0.94 to 0.48 (48.9% decrease) in the presence of Carex. Similar results were observed for NQO-1 expression levels in neuroblastoma cells (Figure 5F). Overall, Carex is a strong suppressor of apoptosis caused by oxidative stress and can be developed as a novel inhibitor of Parkinson’s disease.

## 4. Conclusions

Plant-derived nanovesicles have advantages over EVs produced from cell culture in many aspects, including productivity, safety, production cost, and diversity. In this study, a high amount of Carex was isolated from carrots, using size-exclusion chromatography. Carex, which has properties similar to those of EVs, was further investigated for its antioxidative and apoptotic effects in both cardiomyoblasts and neuroblastoma cells. Carex significantly inhibited ROS generation and apoptosis induction; therefore, the antioxidative effect of Carex can be more effective in the early phase of diseases. Considering its low cytotoxicity, antioxidative function, and high production yield with low cost, Carex is a novel candidate for the therapeutic treatment of myocardial infarction as well as Parkinson’s disease. In this context, the findings of this study will contribute to functional drug development, using nanovesicles from carrots.

## Data Availability

Not applicable.

## References

[1] Snezhkina A.V., Kudryavtseva A.V., Kardymon O.L., Savvateeva M.V., Melnikova N.V., Krasnov G.S., Dmitriev A.A. (2019). ROS Generation and Antioxidant Defense Systems in Normal and Malignant Cells. Oxid. Med. Cell. Longev..

[2] Liguori I., Russo G., Curcio F., Bulli G., Aran L., Della-Morte D., Gargiulo G., Testa G., Cacciatore F., Bonaduce D. (2018). Oxidative stress, aging, and diseases. Clin. Interv. Aging.

[3] Ray P.D., Huang B.W., Tsuji Y. (2012). Reactive oxygen species (ROS) homeostasis and redox regulation in cellular signaling. Cell. Signal..

[4] Lee J.H., Baik J.E., Rhee W.J. (2017). Anti-oxidative effects of silkworm storage protein 1 in HeLa cell. Process Biochem..

[5] Spector A. (2000). Oxidative stress and disease. J. Ocul. Pharmacol. Ther..

[6] Wang M., Wang R., Xie X., Sun G., Sun X. (2019). Araloside C protects H9c2 cardiomyoblasts against oxidative stress via the modulation of mitochondrial function. Biomed. Pharm..

[7] Chen H.X., Liang F.C., Gu P., Xu B.L., Xu H.J., Wang W.T., Hou J.Y., Xie D.X., Chai X.Q., An S.J. (2020). Exosomes derived from mesenchymal stem cells repair a Parkinson’s disease model by inducing autophagy. Cell Death Dis..

[8] Saleem N., Goswami S.K. (2017). Activation of adrenergic receptor in H9c2 cardiac myoblasts co-stimulates Nox2 and the derived ROS mediate the downstream responses. Mol. Cell. Biochem..

[9] Guo J.D., Zhao X., Li Y., Li G.R., Liu X.L. (2018). Damage to dopaminergic neurons by oxidative stress in Parkinson’s disease (Review). Int. J. Mol. Med..

[10] Xu R., Greening D.W., Zhu H.J., Takahashi N., Simpson R.J. (2016). Extracellular vesicle isolation and characterization: Toward clinical application. J. Clin. Investig..

[11] Hartjes T.A., Mytnyk S., Jenster G.W., van Steijn V., van Royen M.E. (2019). Extracellular Vesicle Quantification and Characterization: Common Methods and Emerging Approaches. Bioeng.

[12] Latifkar A., Hur Y.H., Sanchez J.C., Cerione R.A., Antonyak M.A. (2019). New insights into extracellular vesicle biogenesis and function. J. Cell Sci..

[13] Ohno S., Drummen G.P., Kuroda M. (2016). Focus on Extracellular Vesicles: Development of Extracellular Vesicle-Based Therapeutic Systems. Int. J. Mol. Sci..

[14] You J.Y., Kang S.J., Rhee W.J. (2021). Isolation of cabbage exosome-like nanovesicles and investigation of their biological activities in human cells. Bioact. Mater..

[15] Zhang M., Viennois E., Xu C., Merlin D. (2016). Plant derived edible nanoparticles as a new therapeutic approach against diseases. Tissue Barriers.

[16] Rome S. (2019). Biological properties of plant-derived extracellular vesicles. Food Funct.

[17] Patil A.A., Rhee W.J. (2019). Exosomes: Biogenesis, Composition, Functions, and Their Role in Pre-metastatic Niche Formation. Biotechnol. Bioprocess Eng..

[18] Cho S., Yang H.C., Rhee W.J. (2019). Simultaneous multiplexed detection of exosomal microRNAs and surface proteins for prostate cancer diagnosis. Biosens. Bioelectron..

[19] Zhang S., Chuah S.J., Lai R.C., Hui J.H.P., Lim S.K., Toh W.S. (2018). MSC exosomes mediate cartilage repair by enhancing proliferation, attenuating apoptosis and modulating immune reactivity. Biomaterials.

[20] He X., Dong Z., Cao Y., Wang H., Liu S., Liao L., Jin Y., Yuan L., Li B. (2019). MSC-Derived Exosome Promotes M2 Polarization and Enhances Cutaneous Wound Healing. Stem Cells Int..

[21] Lan J., Sun L., Xu F., Liu L., Hu F., Song D., Hou Z., Wu W., Luo X., Wang J. (2019). M2 Macrophage-Derived Exosomes Promote Cell Migration and Invasion in Colon Cancer. Cancer Res..

[22] Jeong K., Yu Y.J., You J.Y., Rhee W.J., Kim J.A. (2020). Exosome-mediated microRNA-497 delivery for anti-cancer therapy in a microfluidic 3D lung cancer model. Lab A Chip.

[23] Yang H.C., Ham Y.M., Kim J.A., Rhee W.J. (2021). Single-step equipment-free extracellular vesicle concentration using super absorbent polymer beads. J. Extracell Vesicles.

[24] Nguyen Cao T.G., Kang J.H., You J.Y., Kang H.C., Rhee W.J., Ko Y.T., Shim M.S. (2021). Safe and Targeted Sonodynamic Cancer Therapy Using Biocompatible Exosome-Based Nanosonosensitizers. ACS Appl. Mater. Interfaces.

[25] Jeong K., Jeong S., Kim J.A., Rhee W.J. (2019). Exosome-based antisense locked nucleic acid delivery for inhibition of type II collagen degradation in chondrocyte. J. Ind. Eng. Chem..

[26] Zhang J., Guan J., Niu X., Hu G., Guo S., Li Q., Xie Z., Zhang C., Wang Y. (2015). Exosomes released from human induced pluripotent stem cells-derived MSCs facilitate cutaneous wound healing by promoting collagen synthesis and angiogenesis. J Transl. Med..

[27] Chen W., Yang M., Bai J., Li X., Kong X., Gao Y., Bi L., Xiao L., Shi B. (2018). Exosome-Modified Tissue Engineered Blood Vessel for Endothelial Progenitor Cell Capture and Targeted siRNA Delivery. Macromol. Biosci..

[28] Bagheri E., Abnous K., Farzad S.A., Taghdisi S.M., Ramezani M., Alibolandi M. (2020). Targeted doxorubicin-loaded mesenchymal stem cells-derived exosomes as a versatile platform for fighting against colorectal cancer. Life Sci..

[29] Kim H., Kang S.J., Rhee W.J. (2021). Phenylboronic Acid-conjugated Exosomes for Enhanced Anticancer Therapeutic Effect by Increasing Doxorubicin Loading Efficiency. Biotechnol. Bioprocess Eng..

[30] Kim H., Rhee W.J. (2020). Exosome-mediated Let7c-5p Delivery for Breast Cancer Therapeutic Development. Biotechnol. Bioprocess Eng..

[31] Kim K., Yoo H.J., Jung J.H., Lee R., Hyun J.K., Park J.H., Na D., Yeon J.H. (2020). Cytotoxic Effects of Plant Sap-Derived Extracellular Vesicles on Various Tumor Cell Types. J. Funct. Biomater..

[32] Özkan İ., Koçak P., Yıldırım M., Ünsal N., Yılmaz H., Telci D., Şahin F. (2021). Garlic (Allium sativum)-derived SEVs inhibit cancer cell proliferation and induce caspase mediated apoptosis. Sci. Rep..

[33] Raimondo S., Naselli F., Fontana S., Monteleone F., Lo Dico A., Saieva L., Zito G., Flugy A., Manno M., Di Bella M.A. (2015). Citrus limon-derived nanovesicles inhibit cancer cell proliferation and suppress CML xenograft growth by inducing TRAIL-mediated cell death. Oncotarget.

[34] Perut F., Roncuzzi L., Avnet S., Massa A., Zini N., Sabbadini S., Giampieri F., Mezzetti B., Baldini N. (2021). Strawberry-Derived Exosome-Like Nanoparticles Prevent Oxidative Stress in Human Mesenchymal Stromal Cells. Biomolecules.

[35] Mu J., Zhuang X., Wang Q., Jiang H., Deng Z.B., Wang B., Zhang L., Kakar S., Jun Y., Miller D. (2014). Interspecies communication between plant and mouse gut host cells through edible plant derived exosome-like nanoparticles. Mol. Nutr. Food Res..

[36] Chen X., Zhou Y., Yu J. (2019). Exosome-like Nanoparticles from Ginger Rhizomes Inhibited NLRP3 Inflammasome Activation. Mol. Pharm..

[37] Ju S., Mu J., Dokland T., Zhuang X., Wang Q., Jiang H., Xiang X., Deng Z.B., Wang B., Zhang L. (2013). Grape exosome-like nanoparticles induce intestinal stem cells and protect mice from DSS-induced colitis. Mol. Ther..

[38] Liu B., Lu Y., Chen X., Muthuraj P.G., Li X., Pattabiraman M., Zempleni J., Kachman S.D., Natarajan S.K., Yu J. (2020). Protective Role of Shiitake Mushroom-Derived Exosome-Like Nanoparticles in D-Galactosamine and Lipopolysaccharide-Induced Acute Liver Injury in Mice. Nutrients.

[39] Zhang M., Viennois E., Prasad M., Zhang Y., Wang L., Zhang Z., Han M.K., Xiao B., Xu C., Srinivasan S. (2016). Edible ginger-derived nanoparticles: A novel therapeutic approach for the prevention and treatment of inflammatory bowel disease and colitis-associated cancer. Biomaterials.

[40] Zhang L., He F., Gao L., Cong M., Sun J., Xu J., Wang Y., Hu Y., Asghar S., Hu L. (2021). Engineering Exosome-Like Nanovesicles Derived from Asparagus cochinchinensis Can Inhibit the Proliferation of Hepatocellular Carcinoma Cells with Better Safety Profile. Int. J. Nanomed..

[41] Donoso-Quezada J., Guajardo-Flores D., Gonzalez-Valdez J. (2020). Enhanced exosome-mediated delivery of black bean phytochemicals (*Phaseolus vulgaris* L.) for cancer treatment applications. Biomed. Pharm..

[42] Cho E.G., Choi S.Y., Kim H., Choi E.J., Lee E.J., Park P.J., Ko J., Kim K.P., Baek H.S. (2021). Panax ginseng-Derived Extracellular Vesicles Facilitate Anti-Senescence Effects in Human Skin Cells: An Eco-Friendly and Sustainable Way to Use Ginseng Substances. Cells.

[43] Webber J., Clayton A. (2013). How pure are your vesicles?. J. Extracell Vesicles.

[44] Nordin J.Z., Lee Y., Vader P., Mager I., Johansson H.J., Heusermann W., Wiklander O.P., Hallbrink M., Seow Y., Bultema J.J. (2015). Ultrafiltration with size-exclusion liquid chromatography for high yield isolation of extracellular vesicles preserving intact biophysical and functional properties. Nanomedicine.

[45] Ramirez M.I., Amorim M.G., Gadelha C., Milic I., Welsh J.A., Freitas V.M., Nawaz M., Akbar N., Couch Y., Makin L. (2018). Technical challenges of working with extracellular vesicles. Nanoscale.

[46] Gurung S., Perocheau D., Touramanidou L., Baruteau J. (2021). The exosome journey: From biogenesis to uptake and intracellular signalling. Cell Commun. Signal..

[47] Xicoy H., Wieringa B., Martens G.J. (2017). The SH-SY5Y cell line in Parkinson’s disease research: A systematic review. Mol. Neurodegener.

